# The Association between Impulsivity and Anxiety in Adolescents with Insomnia: The Moderating Role of Evening Chronotype

**DOI:** 10.1192/j.eurpsy.2025.1184

**Published:** 2025-08-26

**Authors:** Y. L. Wong, H. F. Sit, F. T. W. Cheung, S. X. Li

**Affiliations:** 1 Department of Psychology; 2 The State Key Laboratory of Brain and Cognitive Sciences, The University of Hong Kong, Hong Kong, Hong Kong

## Abstract

**Introduction:**

Adolescence is a unique stage of cognitive, psychosocial and physical growth and development. This period is often associated with increased evening tendency, as well as increased risks for mental health and sleep problems. Insomnia is amongst the most common sleep problem in youths and is commonly associated with impulsivity and anxiety symptoms. Previous research suggested the comorbidity of insomnia and eveningness as a significant risk factor for anxiety symptoms in adolescents. Meanwhile, there has been some evidence suggesting that insomnia and eveningness are respectively associated with impulsivity. Nonetheless, the relationships among eveningness, impulsivity and anxiety in the context of insomnia remained unclear.

**Objectives:**

To investigate how circadian preference moderates the association between impulsivity and anxiety symptoms in adolescents with insomnia.

**Methods:**

Adolescents aged 12-20 years old diagnosed of DSM-V insomnia disorder were recruited. They completed self-reported questionnaires, including the Morningness-Eveningness Questionnaire (MEQ) for assessing circadian preferences, Insomnia severity indexes (ISI) for assessing insomnia symptoms, the General Anxiety Disorder (GAD) scale to measure anxiety symptoms, and the Barret Impulsiveness Scale (BIS) for assessing impulsivity. Participants completed an objective cognitive task (the Balloon Analog Risk Task, BART) to measure risk-taking behavior. Their sleep was objectively assessed by 7-day actigraphy.

**Results:**

Eighty-eight participants were recruited into this study (age:18.20 ± 1.61 years). Among them, 44% of the participants were identified as eveningness type (MEQ < 42). There were no significant differences in anxiety symptoms and impulsivity based on both self-reported and behavioral task between circadian preferences (*all p* > .05). Circadian preferences was found to significantly moderate the association between self-reported impulsivity and anxiety symptoms after controlling age and sex factor (*p* = .009) but not the association between risk-taking behavior and anxiety symptoms (*p* > .05). Specifically, higher self-reported impulsivity was associated with more severe anxiety symptoms in adolescents with insomnia and eveningness (*p* = .006). No significant association was found between self-reported impulsivity and anxiety symptoms in non-eveningness group (*p* > .05).

**Conclusions:**

The findings suggested the role of circadian preference in moderating the association between impulsivity and anxiety symptoms in adolescents with insomnia. Further research may explore different aspects of impulsivity and examine the causation between eveningness, insomnia, impulsivity and anxiety among adolescents in a longitudinal design.

**Disclosure of Interest:**

Y. L. Wong: None Declared, H. F. Sit: None Declared, F. T. W. Cheung: None Declared, S. X. Li Grant / Research support from: This work was funded by General Research Fund (Ref. 17613820), Research Grants Council, University Grants Committee, Hong Kong SAR, China.

